# Detection of Circulating Tumor Cells in Hepatocellular Carcinoma Using Antibodies against Asialoglycoprotein Receptor, Carbamoyl Phosphate Synthetase 1 and Pan-Cytokeratin

**DOI:** 10.1371/journal.pone.0096185

**Published:** 2014-04-24

**Authors:** Jun Li, Lei Chen, Xiaofeng Zhang, Yu Zhang, Huiying Liu, Bin Sun, Linlin Zhao, Naijian Ge, Haihua Qian, Yefa Yang, Mengchao Wu, Zhengfeng Yin

**Affiliations:** 1 Molecular Oncology Laboratory, Eastern Hepatobiliary Surgery Hospital, Second Military Medical University, Shanghai, China; 2 Department of Interventional Radiology, Eastern Hepatobiliary Surgery Hospital, Second Military Medical University, Shanghai, China; The University of Hong Kong, China

## Abstract

**Background:**

Asialoglycoprotein receptor (ASGPR)-ligand-based separation combined with identification with Hep Par 1 or pan-cytokeratin (P-CK) antibody have been demonstrated to detect circulating tumor cells (CTCs) in hepatocellular carcinoma (HCC). The aim of this study was to develop an improved enrichment and identification system that allows the detection of all types of HCC CTCs.

**Methods:**

The specificity of the prepared anti-ASGPR monoclonal antibody was characterized. HCC cells were bound by ASGPR antibody and subsequently magnetically isolated by second antibody-coated magnetic beads. Isolated HCC cells were identified by immunofluorescence staining using a combination of anti-P-CK and anti-carbamoyl phosphate synthetase 1 (CPS1) antibodies. Blood samples spiked with HepG2 cells were used to determine recovery and sensitivity. CTCs were detected in blood samples from HCC patients and other patients.

**Results:**

ASGPR was exclusively expressed in human hepatoma cell line, normal hepatocytes and HCC cells in tissue specimens detected by the ASGPR antibody staining. More HCC cells could be identified by the antibody cocktail for CPS1 and P-CK compared with a single antibody. The current approach obtained a higher recovery rate of HepG2 cells and more CTC detection from HCC patients than the previous method. Using the current method CTCs were detected in 89% of HCC patients and no CTCs were found in the other test subjects.

**Conclusions:**

Our anti-ASGPR antibody could be used for specific and efficient HCC CTC enrichment, and anti-P-CK combined with anti-CPS1 antibodies is superior to identification with one antibody alone in the sensitivity for HCC CTC detection.

## Introduction

Circulating tumor cells (CTCs) are cancer cells shed from either the primary tumor or its metastases that circulate in the peripheral blood. While metastases are directly responsible for the majority of cancer deaths, CTCs may constitute seeds for metastases and may indicate the spread of the disease [1], [2]. Analyses of CTCs hold great promise for the identification of patients at high risk for relapse, the stratification of patients to specific adjuvant therapies, and the monitoring of response to treatment [3]–[5]. So far the epithelial cell adhesion molecule (EpCAM) is widely used to capture CTCs of epithelial origin [6]–[9]. Several EpCAM-targeted methods have been developed and commercially applied for the selection of CTCs including CellSearch system approved by the US Food and Drug Administration (FDA) [10]–[13]. Although the liver is an epithelial organ, the majority of hepatocytes or hepatocellular carcinoma (HCC) cells are EpCAM negative [14]–[17], and the EpCAM-based strategies are not appropriate for detection of HCC CTCs [18] although two studies have recently been conducted to detect EpCAM-positive CTCs as circulating cancer stem cells in patients with HCC [19], [20]. We have previously developed a unique magnetic HCC CTC separation system mediated by the interaction of the asialoglycoprotein receptor (ASGPR) with its ligand [21]. ASGPR is an abundant receptor specific to hepatocytes, recognizes and internalizes glycoproteins that have exposed terminal galactose or N-acetylgalactosamine residues [22], [23]. Given that normal hepatocytes do not circulate, unless they become tumorous, any of the cells detected by the system are circulating HCC cells. However, the ligand-receptor binding assay has its own disadvantages, which will limit its transformation of clinical practice in HCC CTC detection. Since an antibody-antigen binding assay is a better alternative, we prepared a monoclonal antibody specific for ASGPR, modified the magnetic HCC CTC separation method and detection approach, in which HCC CTCs were captured by using anti-ASGPR antibody.

In our previous method, hepatocyte paraffin 1 (Hep Par 1, a human hepatocyte-specific antibody) or pan-cytokeratin (P-CK) antibody alone was used to identify HCC CTCs [21]. The differential expressions of the antigen for Hep Par 1 and CK on the same cell will be the key to ensure that no target cells are missed. Those HCC cells that express the antigen for Hep Par 1 but with low or no CK, or vice versa, may not be identified by a single antibody. To compensate for their low or no expression, we here used a combination of anti-carbamoyl phosphate synthetase 1 (CPS1, a newly identified antigen for Hep Par 1) [24] and anti-P-CK antibodies to allow the detection of all types of HCC CTCs including CPS1^+^/CK^+^, CPS1^−/^CK^+^ and CPS1^+^/CK^−^ HCC cells. The comparison results with the previous method have proven that the current 3-antibody-based method has higher recovery for spiking experiments with tumor cell lines and better CTC detection in blood samples from HCC patients.

## Materials and Methods

### Patients and Sample Collection

The study was approved by the Biomedical Ethics Committee of Eastern Hepatobiliary Surgery Hospital (Shanghai, China) and informed written consent was obtained from all patients. Peripheral blood samples were collected from 27 patients with HCC, 12 with other types of cancer (2 breast cancer, 1 lung cancer, 2 esophageal cancer, 4 gastric cancer and 3 colorectal cancer), 13 with cirrhosis, 5 with chronic hepatitis B, 3 with acute hepatitis A, 2 with chronic hepatitis C, 11 patients with benign intrahepatic space-occupying lesions (5 hepatic hemangioma, 3 liver cysts and 3 focal nodular hyperplasia of the liver), and 15 healthy volunteer. Five milliliters of peripheral blood was drawn from each subject and collected in VACUETTE polyethylene tubes containing EDTA (Greiner Bio-One GmbH, Frickenhausen, Germany). The samples were stored at room temperature and processed within 6 hours after collection except as otherwise indicated. Another 28 liver tissue specimens including HCC (16), cirrhosis (4), fatty liver (1), colorectal liver metastases (2) and normal liver (5) were used for the detection of ASGPR expression.

### Preparation and Characterization of a Mouse Monoclonal Antibody against Human ASGPR

A mouse monoclonal antibody (mAb) against human ASGPR was prepared using the extracellular domain of ASGPR as an immunogen. Briefly, a gene fragment encoding the extracellular domain (a.a. 61–291) of ASGPR1 was generated by RT-PCR amplification and the resulted cDNA was subcloned into prokaryotic vector pGEX-4T-1 (Invitrogen, CA, USA). The recombinant protein was expressed by E. coli BL21 (Invitrogen) and purified for subsequent immunization of female BALB/c mice. The conventional hybridoma technique was used to generate monoclonal antibodies. The isotype and the titer were regularly tested. Inhibition experiment of purified recombinant ASGPR1 protein was conducted to identify the specific binding of the antibody.

### Cell Culture

Human hepatoma cell lines HepG2, Hep3B, Huh7 and PLC/PRF/5, human breast cancer cell line MCF-7, human colon adenocarcinoma cell line SW480, human fibroblast cell line NIH-3T3 and human renal cancer cell line A498 were purchased from American Type Culture Collection and cultured according to ATCC instructions. The cell suspensions were used only when their viability as assessed by trypan blue exclusion exceeded 90%.

### Flow Cytometric Analysis

Primary antibodies were used as follows: the prepared mouse monoclonal antibody of ASGPR, mouse anti-CPS1 antibody (Abcam, MA, USA), mouse anti-P-CK antibody CK3-6H5 (Miltenyi Biotec GmbH, Bergisch Gladbach, Germany), or an antibody cocktail of CPS1 and P-CK. For each staining, a total of 4×10^5^ cells were incubated with primary antibody at 37°C for 45 minutes, followed by incubation with fluorescein isothiocyanate (FITC)-conjugated secondary antibody (Beyotime, Shanghai, China) at 4°C for 30 minutes in the dark. Flow cytometric analysis was carried out on FACSCalibur (Becton Dickinson, CA, USA), and the obtained data were analyzed with FlowJo software Version 7.6.1 (Tree Star Inc., OR, USA).

### Immunofluorescence Laser Confocal Analysis

HepG2 cells were fixed with 4% formaldehyde for 15 minutes at room temperature and permeabilized with 0.04% Triton X-100. Then the cells were incubated with anti-ASGPR mAb at 4°C overnight followed by FITC-conjugated goat anti-mouse IgG antibody at room temperature for 30 minutes in the dark, and costained with 4′,6-diamidino-2-phenylindole (DAPI; Sigma, MO, USA). Cell imaging was performed in the multitracking mode on a laser scanning confocal microscope LSM510 (Carl Zeiss, Oberkochen, Germany) using a sequential scan setting with excitation at 359 and 490 nm. Emission was collected at 461 nm (DAPI) and 520 nm (FITC).

### Immunohistochemistry

For non-fluorescent immunohistochemistry, the tissue sections were incubated with anti-ASGPR mAb at 4°C overnight followed by a horseradish peroxidase (HRP)-conjugated secondary antibody (Maixin-Bio, Fuzhou, China) at room temperature for 45 minutes, and the immunoreactivity was detected utilizing diaminobenzidine (DAB) substrate (Maixin-Bio). For triple-fluorescent immunohistochemistry, the tissue sections were incubated with mouse anti-P-CK antibody and rabbit anti-CPS1 antibody at 4°C overnight followed by staining with Cy3-conjugated goat anti-mouse IgG antibody and FITC-conjugated goat anti-rabbit IgG antibody (Beyotime) and costaining with DAPI at room temperature for 30 minutes.

### Mononuclear Cell Enrichment Followed by Magnetic Separation

Mononuclear cells and tumor cells were enriched from the whole blood samples by density gradient Ficoll-Paque PLUS (GE Healthcare, WI, USA) according to previously described method [21]. For magnetic labeling, enriched cells were incubated with anti-ASGPR mAb for 1 hour at 37°C. After washed with dilution buffer, cells were incubated with anti-Mouse IgG1 MicroBeads (Miltenyi Biotec GmbH, Bergisch Gladbach, Germany) for 15 minutes at 4°C. Magnetically labeled cells were isolated over the AutoMACS Pro Separator (Miltenyi Biotec GmbH) with “posseld2” program. The positive fraction was spun down on a polylysine-coated slide using a cytocentrifuge (Wescor Inc., UT, USA) and the slide was fixed in 4% formaldehyde followed by immunoflourescence staining.

### Immunofluorescence Staining

A mouse mAb cocktail against CPS1 and P-CK and a rat anti-human CD45 monoclonal antibody (Santa Cruz, CA, USA) were used as primary antibodies. After blocking nonspecific binding sites, slides were incubated for 1 hour with primary antibodies at 37°C. A Cy3-conjugated goat anti-mouse IgG antibody and an Alexa Fluor 488-conjugated rabbit anti-rat IgG antibody (Invitrogen) served as secondary antibodies. After DAPI staining to visualize nuclei, the slides were mounted in antifade solution (Beyotime) and viewed through a fluorescence microscope Olympus IX71 (Olympus, Tokyo, Japan).

### Identification and Enumeration of CTCs

The slides were imaged according to previously described method [21]. Captured images were carefully examined and the objects that met preset criteria were counted. CTC counts were expressed as the number of cells per 5 mL of blood.

### Spiking Experiments with Tumor Cell Lines

Briefly, 5-mL aliquots of peripheral blood from healthy adults were collected into VACUETTE polyethylene tubes containing EDTA, spiked with various numbers of HepG2 cells or MCF-7 cells, and then processed as described above.

### Statistical Analysis

Continuous variables are presented as mean±SD. Statistical significance was calculated by Student’s t-test. All statistical analyses were carried out with the SPSS statistical software package (SPSS Inc., IL, USA). A 2-sided *P*<0.05 was considered statistically significant.

## Results

### ASGPR was Exclusively Expressed in Human Hepatoma Cell Line, Normal Hepatocytes and HCC Cells in Tissue Specimens Detected by the ASGPR Antibody Staining

**Figure 1:**
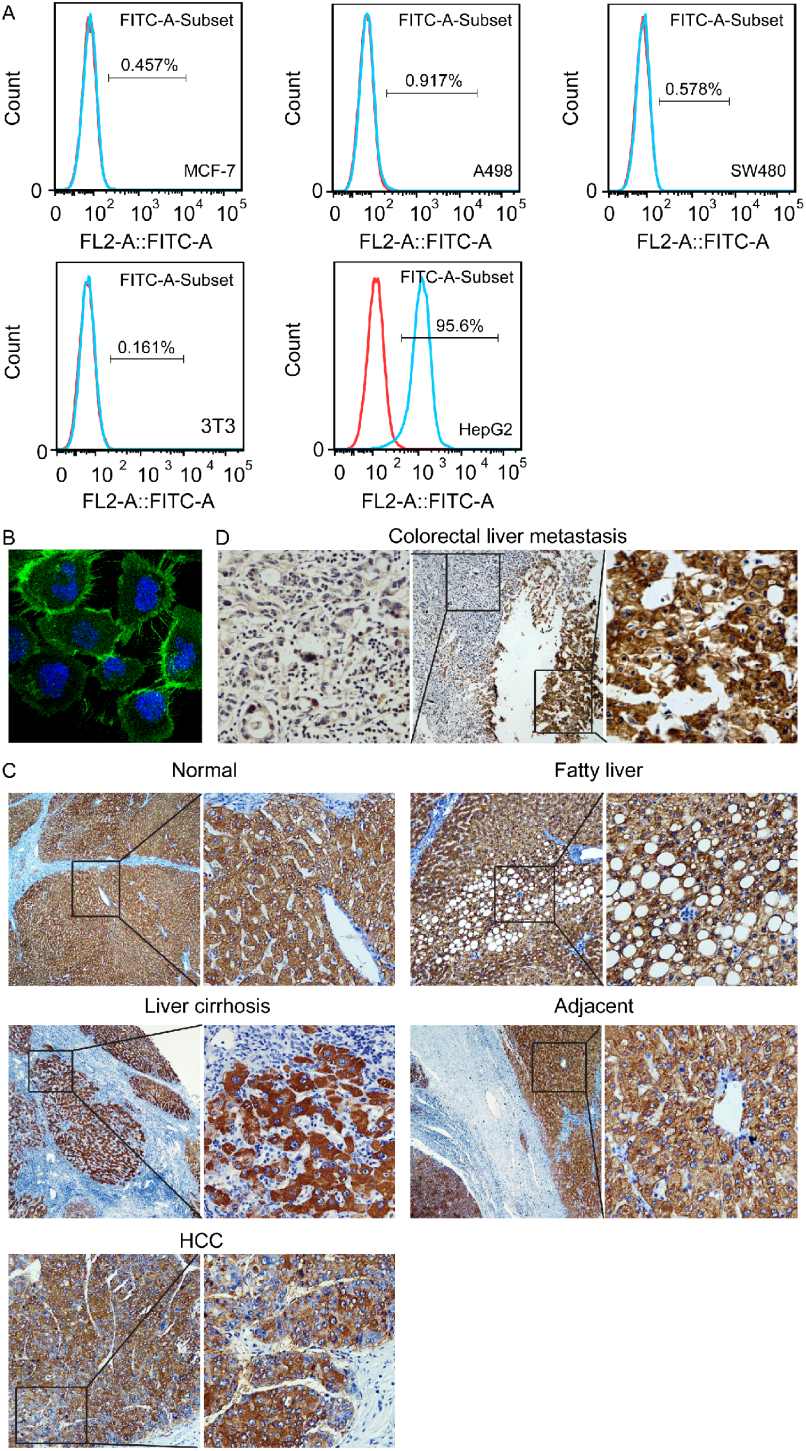
Expression of ASGPR detected by the prepared mouse monoclonal antibody. (A) ASGPR was exclusively expressed analyzed by flow cytometry in human hepatoma cell line HepG2. (B) The antibody-binding site was localized on the outer cell membrane in HepG2 cells visaulized by confocal laser scanning microscopy. (C) ASGPR was expressed determined by immunohistochemistry at the membrane of the hepatocytes and HCC cells in liver tissue, HCC tissue and adjacent hepatic tissue (magnification, ×200). D, Immunohistochemical staining of ASGPR clearly differantiated between hepatic (right) and extrahepatic (left) tissues in a liver metastatic tissue from a colon carcinoma (magnification, ×200).

The binding of the anti-ASGPR mAb to a single cell of cell lines from various origins was firstly analyzed by flow cytometry. The results showed that all the test cell lines derived from extrahepatic origin including MCF-7, A498, SW480, and NIH-3T3 didn’t bind the antibody (Fig. 1A), but on the contrary, the expression level of ASGPR can be measured in the human hepatoma cell line HepG2 (Fig. 1A), and the positive expression rate was (95.1±2.6)%. Subsequently, the antibody-binding site was visualized by confocal laser scanning microscopy in HepG2 cells, and strong positive staining for ASGPR was observed on the cell surface (Fig. 1B), indicating that the antigen recognized by the antibody is present in the outer cell membrane.

In normal liver tissue, HCC tissue, and adjacent hepatic tissue, immunostaining of ASGPR was observed at the membrane of the hepatocytes and HCC cells (Fig. 1C), but several extrahepatic tissues examined (colon, lung, renal carcinoma, and breast cancer) failed to be stained by the same procedure (data not shown). As shown in Fig. 1D, immunostaining in normal liver tissue adjacent to metastatic lesion of a colon carcinoma could clearly differentiate between hepatic and extrahepatic tissues. Although the staining intensity in various HCC tissues could be uniform, all the 16 HCC tissues examined exhibited staining for the membranes of HCC cells (Fig. 1C). These results suggest that the anti-ASGPR mAb might be used for magnetic cell separation to capture primary tumor cells in clinical HCC specimens.

### More HCC Cells could be Identified by the Antibody Cocktail for CPS1 and P-CK Compared with a Single Antibody

**Figure 2:**
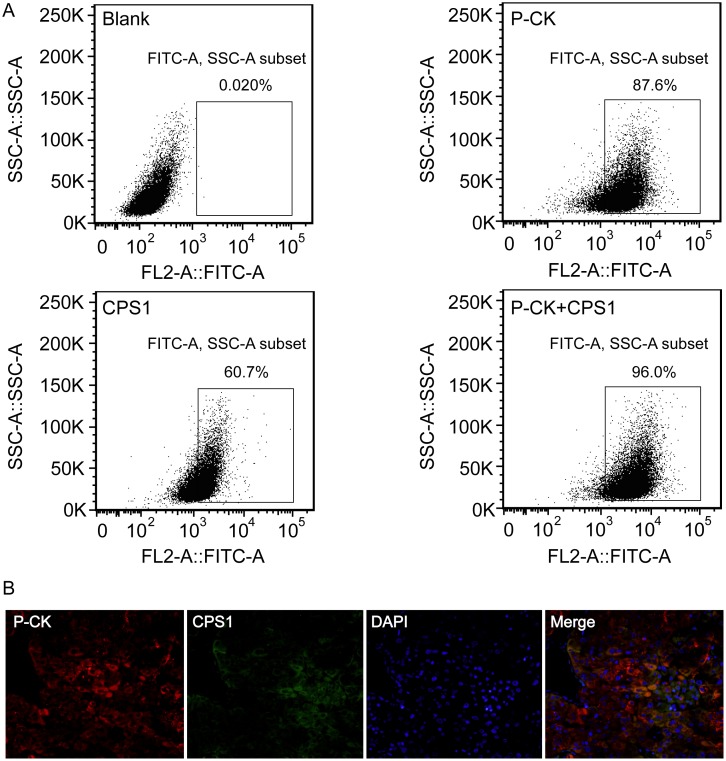
More HCC cells could be identified by the antibody cocktail for CPS1 and P-CK than a single antibody. (A) Positive rates of immunostaining for anti-P-CK combined with anti-CPS1 antibodies in PLC/PRF/5 cells were increased examined by flow cytometry compared with those for a single antibody. (B) Triple immunofluorescence staining with antibodies against P-CK (red), CPS1 (green) and DAPI (blue) in a HCC specimen (magnification, ×200). A few HCC cells didn’t stain positive for CPS1 or P-CK, but almost all of HCC cells stained positive for both.

The expressions of CPS1 and P-CK in various hepatoma cell lines were firstly analyzed by flow cytometry. Either CPS1 or P-CK indicated different expression rates in various cell lines examined, ranging between 10% and 99%. PLC/PRF/5 cell line, which had relatively low expressions of both CPS1 and P-CK, was then examined by using the antibody cocktail for CPS1 and P-CK, and more cells could be identified, expression rates increasing from (62.1±7.6)% and (85.2±4.4)% respectively to (96.7±2.1)% (Fig. 2A). Subsequently, the examination of CPS1 and P-CK expressions was further extended to human HCC specimens, both of them were simultaneously determined in one specimen by three color immunofluorescence staining. As shown in Fig. 2B, a few HCC cells didn’t stain positive for CPS1 or P-CK although almost all of HCC cells stained positive for both. All these results suggest that some tumor cells express low or no CPS1 or P-CK, staining with CPS1 or P-CK antibody alone may miss some HCC cells, and anti-CPS1 combined with anti-P-CK antibodies may identify more HCC cells than a single antibody.

### Recovery and Specificity of HCC CTC Detection

Different numbers of HepG2 cells were spiked into blood, and recovery was measured by the methods as described above. The average recovery of HepG2 cells was 80% or more at each spiking level (Table 1). No tumor cells were detected in any samples spiked with cell lines MCF-7 and A498 (200 cells spiked). For the method comparison study, EDTA or heparin blood spiked with 200 HepG2 cells were maintained at room temperature for different time intervals (0 hour, 24 hours, 48 hours or 72 hours) and processed within a maximum of 72 hours after blood drawing. The current antibody-based approach obtained a higher recovery rate of HepG2 cells at each time interval than the ligand-based approach (Table 2).

**Table 1:** Accuracy of the current system analyzed by recovery of HepG2 cells spiked into blood.

Spiked cell number	Detected cell number	Recovery (%)
10	8±1	80±7
50	43±3	85±5
250	211±7	84±3
1000	824±19	82±2

**Table 2:** A comparison of HepG2 cell recovery between the antibody-based approach and the ligand-based approach at different time intervals.

Time intervals	The antibody-based approach		The ligand-based approach		*P*
	Detected cell number	Recovery (%)	Detected cell number	Recovery (%)	
0 hour	163±9	82±5	131±15	66±8	0.035
24 hours	161±10	81±5	110±21	55±11	0.019
48 hours	153±15	77±8	77±18	39±9	0.005
72 hours	136±28	68±14	38±24	19±12	0.010

NOTE: In the antibody-based approach, all observed differences between CTC values at 0 hour and after storage were not statistically significant (Student’s t-test).

### Detection of CTCs in Blood Samples from HCC Patients and other Human Beings

**Figure 3:**
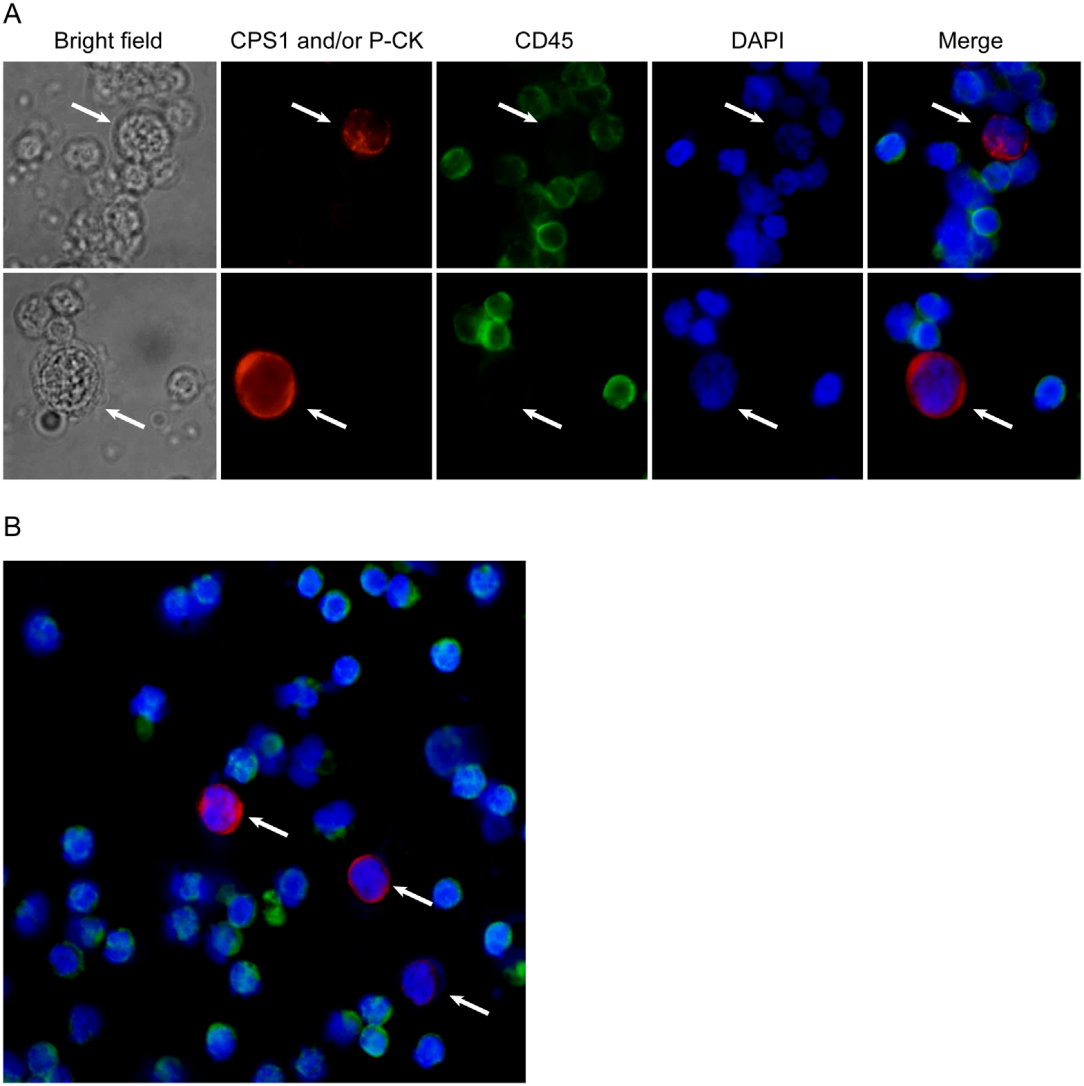
CTCs (indicated by arrow) detected in blood from patients with HCC by the current 3-antibody-based method (magnification, ×200). (A) A large cell with a morphologically intact DAPI-stained nucleus (blue), CPS1 or/and P-CK (red) positive and CD45 (green) negative was considered a HCC CTC. (B) Several CTCs observed in a same field of view.

The criteria for HCC CTC determination are considered to be a large cell with a morphologically intact DAPI-stained nucleus, CPS1 or/and P-CK positive and CD45 negative (Fig. 3). CTCs were detected in 24 of 27 (89%) patients with HCC, and the results were shown in Table S1. The number of CTCs detected ranged from 0 to 102 per 5 mL, with an average of 34±27. The correlations between CTC numbers and clinical variables of HCC patients were summarized in Table 3. On the contrary, no CTCs were detected in 15 healthy volunteers, 13 with cirrhosis, 5 with chronic hepatitis B, 2 with chronic hepatitis C, 3 with acute hepatitis A, 11 with benign intrahepatic space-occupying lesions. In the samples from 12 patients with other types of advanced cancer, no CTCs were also detected.

**Table 3:** Summary of CTC detection in 27 patients with HCC.

Clinical variable	No. of patients	Mean±SD	*P*
**Age(years)**			0.550
≤50	14	31±28	
>50	13	37±27	
**Gender**			0.667
Male	21	32±27	
Female	6	38±31	
**Tumor Size(cm)**			<0.001
<5	18	20±16	
>5	9	62±23	
**Portal vein tumor thrombus**			<0.001
With	11	61±20	
Without	16	15±11	
**TNM Staging**			<0.001
I–II	15	14±11	
III–IV	12	58±22	

### More CTCs could be Detected from HCC Patients by the Current 3-antibody-based Method Compared with the Previous Method

The CTC detection efficiency of the current 3-antibody-based method and the previous method was compared using the same blood samples from HCC patients. The number of CTCs detected in the same blood samples from 10 patients with HCC using two methods was shown in Table 4. As can be seen in this table, higher CTC counts were detected in all patients examined by the current method (*P* = 0.001). In all 6 HCC patients with more than 40 CTCs, 14–26% higher sensitivity (20% averaged) of CTC detection was consistently achieved. These results confirmed that this new strategy could increase the sensitivity for CTC detection in HCC patients.

**Table 4:** **A** comparison of CTC detection in a same patient with HCC between the current 3-antibody-based method and the previous method.

Patient No.	Detected CTCs numbers		Sensitivity advanced (%)
	Previous method	Current method	
5	19	21	11
8	68	86	26
10	21	24	14
11	25	29	16
12	57	70	23
15	45	54	20
16	43	49	14
18	31	37	19
21	49	59	20
27	43	51	19

## Discussion

We have previously developed a unique magnetic HCC CTC separation system mediated by the interaction of ASGPR with its ligand [21]. However, the ligand-receptor binding assay has its own limitations: (1) Since the reaction is dependent on calcium ions, sodium citrate and EDTA can’t be used as anticoagulants for the blood sample, and only heparin will serve as an anticoagulant. Almeida et al. have found that addition of heparin may cause gelling of cell suspensions in the purification of lymphocytes [25]. We have also noted that mononuclear cell suspensions purified from whole human blood in heparin slowly flow over a magnetic separator, probably due to gel formation, which may affect CTC separation efficiency. (2) The HCC CTCs to be assayed must be living cells, which requires more rigorous methods to allow for specimen collection, preservation, transportation, and processing. (3) Cell surface receptor activity is controlled by various microenvironmental factors, for example, calcium could induce a conformational change in the ligand binding domain of the receptor, and pH may regulate receptor function by altering the amount of calcium bound to the receptor [26]–[28].

All these drawbacks might be avoided or reduced by an antibody-antigen binding approach. So we used the extracellular domain of ASGPR produced by genetically engineered bacterial cells as an antigen, and prepared a monoclonal antibody specific for ASGPR. The test results indicated that the antibody can tag ASGPR-positive cells with high affinity, but can’t bind the non-liver-derived cells. In order to demonstrate the availability of the ASGPR antibody in HCC CTC capture, ASGPR expressions were examined in human cancer cell line and HCC tissues by using flow cytometry and immunohistochemistry. All the tested human non-liver tumor cell lines showed negative ASGPR expression. However, almost all the HCC tissues and primary human HCC cells examined showed ASGPR positive expression, in sharp contrast, non-liver tissues and liver metastases from colorectal cancer showed ASGPR negative expression, indicating the availability of the ASGPR antibody in HCC CTC capture.

We next modified the magnetic HCC CTC separation method and compared it with our previous method. The antibody-based approach can capture more cells at each time interval than the ligand-based approach, circumventing, at least partly, the above-mentioned intrinsic limitations of the ligand-based approach. In other words, the former could test long-term stored samples, which allows for specimen preservation and transportation.

In the current method, the separated HCC CTCs were subsequently identified by immunofluorescence after staining with a triple stain procedure, wherein positive staining for CPS1, P-CK and DAPI, negative staining for CD45 is indicative of HCC CTCs. CPS1 is a liver specific, intramitochondrial, rate-limiting enzyme in the urea cycle [29]. A previous study showed that CPS1 is the antigen for Hep Par 1, a commonly used antibody in diagnostic surgical pathology practice to determine the hepatocellular origin of neoplasms [24]. Our experiment indicated that anti-CPS1 antibody was more appropriate for identification of HCC CTCs than Hep Par 1 (data not shown). However, several authors reported the heterogeneous expression of CPS1 in human HCC by using CPS1 antibody or Hep Par 1 [30], [31]. It means that staining with CPS1 antibody alone may miss some HCC CTCs that lack expression of CPS1. P-CK is the epithelial-specific tumor-associated marker signature, and has been widely used for the identification of CTCs from peripheral blood mononuclear cells [32]–[34]. It is noteworthy that disseminating tumor cells can undergo the epithelial to mesenchymal transition (EMT), which can result in at least partial downregulation of epithelial cell-specific molecules including CK [35]–[37], and this transition may endow tumor cells with stem cell properties enabling self-renewal [38]. Thus, the CTC technologies based only on epithelial markers such as CK-based methods might be “blind” to potentially the most dangerous CTCs [39], [40]. Alternatively, a mixture of two antibodies against CPS1 and P-CK may be optional. When the anti-CPS1 antibodies are added to the CK detection process, it will be a plus effect. In fact, our results revealed that some HCC cells really stained negative for CPS1 or P-CK, and more HCC cells could be identified by the antibody cocktail compared with a single antibody. Based on the results, it can be imagined that the detection of HCC CTCs with the combined antibodies may increase the sensitivity and reproducibility by the compensation effect of biomarkers from each other, and minimize the possible false negative results.

By using this system, recovery of HepG2 cells was greater than 80% at different spiking level and no tumor cells were found in all blood samples spiked with breast cancer cells. Importantly, the parallel tests for blood samples spiked with HepG2 cells have demonstrated that the current system could detect more HepG2 cells than the previous system. For blood tests, CTCs were not detected in healthy volunteers, individuals with non-HCC liver diseases, and patients with non-HCC advanced cancers, but could be detected in HCC patients. Moreover, a higher CTC count was detected in almost all patients examined by the current system compared to the previous system. In other words, the previous assay process could lose a portion of the real CTC population in individual patients. Collectively, all these results indicate the high specificity and sensitivity of the current system, which may be beneficial for the isolation and detection of HCC CTC clusters.

In summary, we have improved a higher specific and sensitive system with three antibodies for CTC detection in HCC patients, which will allow for accurate enumeration and subsequent molecular analysis of individually isolated HCC CTCs as a prognostic marker or a therapeutic target.

## Supporting Information

Table S1 Clinicopathologic profiles and detection of CTCs in patients with HCC.
(DOC)
