# Supplementary material for: Detection of Circulating Tumor Cells in Hepatocellular Carcinoma Using Antibodies against Asialoglycoprotein Receptor, Carbamoyl Phosphate Synthetase 1 and Pan-Cytokeratin
Source: PLoS One. 2014 Apr 24;9(4):e96185. doi: 10.1371/journal.pone.0096185 (PMC3999270; doi:10.1371/journal.pone.0096185)
Supplement: Table S1 — Clinicopathologic profiles and detection of CTCs in patients with HCC. (DOC) [file pone.0096185.s001.doc]

**Supplementary table S1. Clinicopathologic profiles and detection of CTCs in patients with HCC**

| Patient No. | Age/Gender | Tumor size(cm) | Portal vein tumor thrombus | TNM^a^ | CTCs |
| --- | --- | --- | --- | --- | --- |
| 1 | 45/M | >5 | + | Ⅲ | 41 |
| 2 | 61/M | <5 | - | Ⅱ | 17 |
| 3 | 42/F | <5 | - | Ⅰ | 7 |
| 4 | 32/M | <5 | - | Ⅰ | 0 |
| 5 | 41/M | <5 | - | Ⅱ | 21 |
| 6 | 55/M | <5 | - | Ⅱ | 13 |
| 7 | 71/M | >5 | + | Ⅲ | 45 |
| 8 | 65/M | >5 | + | Ⅳ | 86 |
| 9 | 28/F | >5 | - | Ⅲ | 27 |
| 10 | 42/M | <5 | - | Ⅱ | 24 |
| 11 | 58/M | <5 | - | Ⅱ | 29 |
| 12 | 50/F | >5 | + | Ⅲ | 70 |
| 13 | 48/M | <5 | - | Ⅰ | 11 |
| 14 | 76/M | <5 | - | Ⅱ | 19 |
| 15 | 62/M | >5 | + | Ⅲ | 54 |
| 16 | 54/M | <5 | + | Ⅲ | 49 |
| 17 | 49/M | <5 | - | Ⅰ | 0 |
| 18 | 34/M | <5 | + | Ⅲ | 37 |
| 19 | 36/M | <5 | - | Ⅱ | 13 |
| 20 | 64/M | <5 | - | Ⅰ | 5 |
| 21 | 61/M | >5 | + | Ⅲ | 59 |
| 22 | 70/F | >5 | + | Ⅲ | 73 |
| 23 | 48/M | >5 | + | Ⅳ | 102 |
| 24 | 57/F | <5 | - | Ⅰ | 0 |
| 25 | 47/M | <5 | - | Ⅱ | 24 |
| 26 | 73/M | <5 | - | Ⅱ | 32 |
| 27 | 38/F | <5 | + | Ⅲ | 51 |

^a^Sixth edition of International Union Against Cancer (UICC) tumor-node-metastasis (TNM) staging system of HCC (2002).
